# Conserved localization of Pax6 and Pax7 transcripts in the brain of representatives of sarcopterygian vertebrates during development supports homologous brain regionalization

**DOI:** 10.3389/fnana.2014.00075

**Published:** 2014-08-06

**Authors:** Nerea Moreno, Alberto Joven, Ruth Morona, Sandra Bandín, Jesús M. López, Agustín González

**Affiliations:** Department of Cell Biology, Faculty of Biology, Complutense University of MadridMadrid, Spain

**Keywords:** Pax genes, immunohistochemistry, segmental organization, telencephalon, diencephalon, mesencephalon, brain evolution

## Abstract

Many of the genes involved in brain patterning during development are highly conserved in vertebrates and similarities in their expression patterns help to recognize homologous cell types or brain regions. Among these genes, Pax6 and Pax7 are expressed in regionally restricted patterns in the brain and are essential for its development. In the present immunohistochemical study we analyzed the distribution of Pax6 and Pax7 cells in the brain of six representative species of tetrapods and lungfishes, the closest living relatives of tetrapods, at several developmental stages. The distribution patterns of these transcription factors were largely comparable across species. In all species only Pax6 was expressed in the telencephalon, including the olfactory bulbs, septum, striatum, and amygdaloid complex. In the diencephalon, Pax6 and Pax7 were distinct in the alar and basal parts, mainly in prosomeres 1 and 3. Pax7 specifically labeled cells in the optic tectum (superior colliculus) and Pax6, but not Pax7, cells were found in the tegmentum. Pax6 was found in most granule cells of the cerebellum and Pax7 labeling was detected in cells of the ventricular zone of the rostral alar plate and in migrated cells in the basal plate, including the griseum centrale and the interpeduncular nucleus. Caudally, Pax6 cells formed a column, whereas the ventricular zone of the alar plate expressed Pax7. Since the observed Pax6 and Pax7 expression patterns are largely conserved they can be used to identify subdivisions in the brain across vertebrates that are not clearly discernible with classical techniques.

## INTRODUCTION

Brain development is currently analyzed under the novel perspective of “genoarchitectonics,” which refers to the neural expression of genes coding proteins activated or repressed in spatially restricted patterns regulated by genomic regulatory regions (Puelles and Ferran, 2012). This is the case of the Pax gene family that possesses important roles in development and has arisen from the duplication of a single ancestral gene and/or chromosome during the early history of metazoans.

Pax genes encode a family of highly conserved transcription factors characterized by the presence of a paired domain that confers sequence-specific binding to DNA; in addition, Pax transcription factors may also have an octapeptide motif and part or all of a homeobox DNA-binding domain (Balczarek et al., 1997; Chi and Epstein, 2002; Vorobyov and Horst, 2006; Lang et al., 2007; Wang et al., 2010). This family shows a high degree of evolutionary conservation throughout diverse lineages of metazoans, making it an ideal system to address relationships inside chordate phylum. Nine Pax family members (1–9) were identified in vertebrates, which encode proteins exhibiting highly conserved structure, genomic organization, expression patterns, and biological functions. The members of the Pax gene family are grouped into four classes according to their structure and paired domain homology, I (Pax1/9), II (Pax2/5/8), III (Pax3/7), and IV (Pax4/6), that arose before the divergence of *Drosophila* and vertebrates, i.e. prior to the Cambrian radiation of triploblastic metazoan body plan. The genes and the regulatory sequences evolved by the precise DNA duplicating machinery, emerging their expression patterns, enabling the animal survival in the environmental conditions (Puelles and Ferran, 2012). Functional analysis indicates that Pax genes act singularly and not in combination, in contrast to other gene families such as the Hox gene family (Kessel and Gruss, 1990). However, in spite of the evolutionary conservation of the different Pax members, there are still important questions to be answered, such as to what extent are all of the molecularly conserved genes expressed in homologous structures? We can shed light on this question taking into account both sequence comparison and genoarchitectonic analysis.

The neural tube of all vertebrates possesses multiple subdivisions along the rostrocaudal and dorsoventral axis, each being characterized by a specific combination of developmental regulatory genes (Puelles and Rubenstein, 2003). In this regard, the analysis of the Pax genes expression patterns is most relevant because it is a very conserved family in terms of genetic structure and function and, in addition, all the members that are expressed in the brain show very conserved expression patterns in all vertebrates analyzed. Moreover, it has been often noted that they have a biphasic function, first in brain regionalization and later in cell specification (reviewed in Blake et al., 2008). Therefore, the study of these genes suits evolutionary and comparative analysis of brain organization. In these same comparative studies it is possible to analyze the topological organization and the specific cell groups that are produced in each brain subdivision.

Among the Pax genes, Pax6 and Pax7 are expressed in regionally restricted patterns in the developing brain and are involved in neuronal proliferation, brain regionalization, cell differentiation, and neuronal survival (Wehr and Gruss, 1996; Lang et al., 2007; Thompson et al., 2007; Osumi et al., 2008; Wang et al., 2008). Interestingly, Pax6 and Pax7 are also expressed in adult brains in restricted and well-localized cell groups and regions (Walther and Gruss, 1991; Stoykova and Gruss, 1994; Kawakami et al., 1997; Shin et al., 2003; Thompson and Ziman, 2011; Duan et al., 2012), suggesting their involvement in the maintenance of distinct neuronal identity (Ninkovic et al., 2010), in physiological functions in mature neurons (Stoykova and Gruss, 1994; Shin et al., 2003), and as key regulators of a cell’s measured response to a dynamic environment (Blake et al., 2008).

In the present account, we have analyzed the expression patterns of Pax6 and Pax7 in the brain of representative species of tetrapods, including amniote (reptiles, *Pseudemys scripta;* birds, *Gallus gallus*; mammals, *Mus musculus*) and anamniote (anuran amphibian, *Xenopus laevis*; urodele amphibian, *Pleurodeles waltl*) vertebrates. In addition, data are presented for the first time on the distribution of these transcription factors in the brain of lungfishes (*Neoceratodus forsteri*), the closest living relatives of tetrapods (Brinkmann et al., 2004; Chen et al., 2012; Amemiya et al., 2013). We selected Pax6 and Pax7 because, as previously mentioned, several studies in different species had previously shown that they are widely expressed in distinct brain regions. However, in most of those studies only fragmentary data were reported about the neuroanatomical distribution of these transcription factors. The aim of the present report was to provide comparative information on the sequence and expression patterns for Pax6 and Pax7 across a vast group of vertebrates to show shared and distinct features across taxa. Data on selected developmental stages and juveniles of the different species used are presented. Given the difficulty of conducting genetic approaches to the study of the neuroanatomy of so many species, we will rely on immunohistochemical techniques that use antibodies against the transcription factors, which are largely conserved. The validity of this approach has been reinforced by the localization of these transcription factors that, in addition to being expressed during development, are also found in adult animals; their distribution serves as a tool for recognizing brain regionalization, particularly those many entities that are not cytoarchitectonically distinct (González and Northcutt, 2009; Moreno et al., 2010, 2012b; Ferreiro-Galve et al., 2012; Bandín et al., 2013; Joven et al., 2013a,b). The results of this comparative analysis highlight that the expressions of Pax6 and Pax7 are highly conserved within the whole group of sarcopterygians (lungfishes and tetrapods) suggesting a similar role of these genes in the regionalization of the brain and the specification of corresponding cell populations. Furthermore, the expression of these genes may help defining homologous brain regions in many other species.

## MATERIALS AND METHODS

### ANIMALS AND TISSUE PROCESSING

For the present study adult, juvenile, and developing specimens were used. Brains analyzed were of mice (*M. musculus*; developmental stages included embryonic and postnatal stages), chickens (*G. gallus*, classified according to Hamburger and Hamilton, 1951; 8–11 days or stages HH33–HH37 were used), turtles (*Pseudemys scripta*, prehatching; 1–2 weeks prehatching and less than 5 cm long specimens were used), frogs (*X. laevis*, embryonic and larval stages were used, classified according to Nieuwkoop and Faber, 1967), newts (*Pleurodeles waltl*, embryonic and larval stages were used, classified according to Gallien and Durocher, 1957), and lungfish (*N. forsteri*; developmental staging according to Kemp, 1982; stages 45–46 were used). All animals were treated according to the regulations and laws of the European Union (2010/63/UE) and Spain (Royal Decrees 53/2013) for care and handling of animals in research and after approval from the Complutense University to conduct the experiments described. The lungfish material was sent fixed by Dr. Jean M. P. Joss (Biological Sciences, Macquarie University, Sydney, NSW 2109, Australia) and we have conducted the staining in Madrid. For all the other species, at appropriate times, animals were deeply anesthetized and fixed by immersion or perfusion in cold 4% paraformaldehyde in a 0.1 M phosphate buffer (PB, pH 7.4). The brains were removed and kept in the same fixative for 2–3 h. Subsequently, they were immersed in a solution of 30% sucrose in PB for 4–6 h at 4°C until they sank, embedded in a solution of 20% gelatin with 30% sucrose in PB, and stored for 6 h in a 10% formaldehyde solution at 4°C. The brains were cut on a freezing microtome at 20–30 μm in the transverse, horizontal or sagittal plane, and sections were collected and rinsed in cold PB.

### WESTERN BLOTTING

The animals were anesthetized and the brains were quickly removed and mechanically homogenized in an equal volume of cold buffer (5 mM EDTA, 20 mM Tris, pH 7.4, 150 mM NaCl, 10% glycerol, 1% Nonidet P40; Roche) supplemented with protease and phosphatase inhibitors (50 μg/ml phenylmethylsulfonyl fluoride, 10 μg/ml aprotinin, 25 μg/ml leupeptin, and 100 nM orthovanadate; all from Sigma, St. Louis, MO, USA). Samples containing 50 μg of protein were applied in each lane of a 12% polyacrylamide gel (161-0801; Bio-Rad, Hercules, CA, USA) and separated by sodium dodecyl sulfate-polyacrylamide gel electrophoresis (SDS-PAGE) with a Mini-Protean system (Bio-Rad, Hercules, CA, USA). The samples of rat brain and molecular weight standards (Precision Plus Protein Dual Color Standards; Bio-Rad, Hercules, CA, USA) were run in other lanes. The separated samples in the gel were transferred to a nitrocellulose membrane (Bio-Rad, Hercules, CA, USA). Nonspecific binding sites were blocked by incubation overnight in Tris–HCl buffer (TBS) containing 0.1% Tween-20 and 5% nonfat milk, at 4°C. The blots were then incubated for 24 h at 4°C in primary antibody dilution (as for immunohistochemistry). After rinsing in TBS, the blots were incubated in horseradish peroxidase-coupled secondary goat anti-mouse or goat anti-rabbit antisera (Jackson Immunoresearch, West Grove, PA, USA; diluted 1:15,000) for 2 h at room temperature. Immunoreactive bands were detected by using an enhanced chemiluminescence system (Super Signal West Pico Chemiluminiscent Substrate; Pierce, Thermo Scientific, Rockford, IL, USA). Photographs were taken after applying an autoradiographic film to the membrane, in darkness, for 1–4 min.

### SEQUENCE ANALYSIS

**FIGURE 1:**
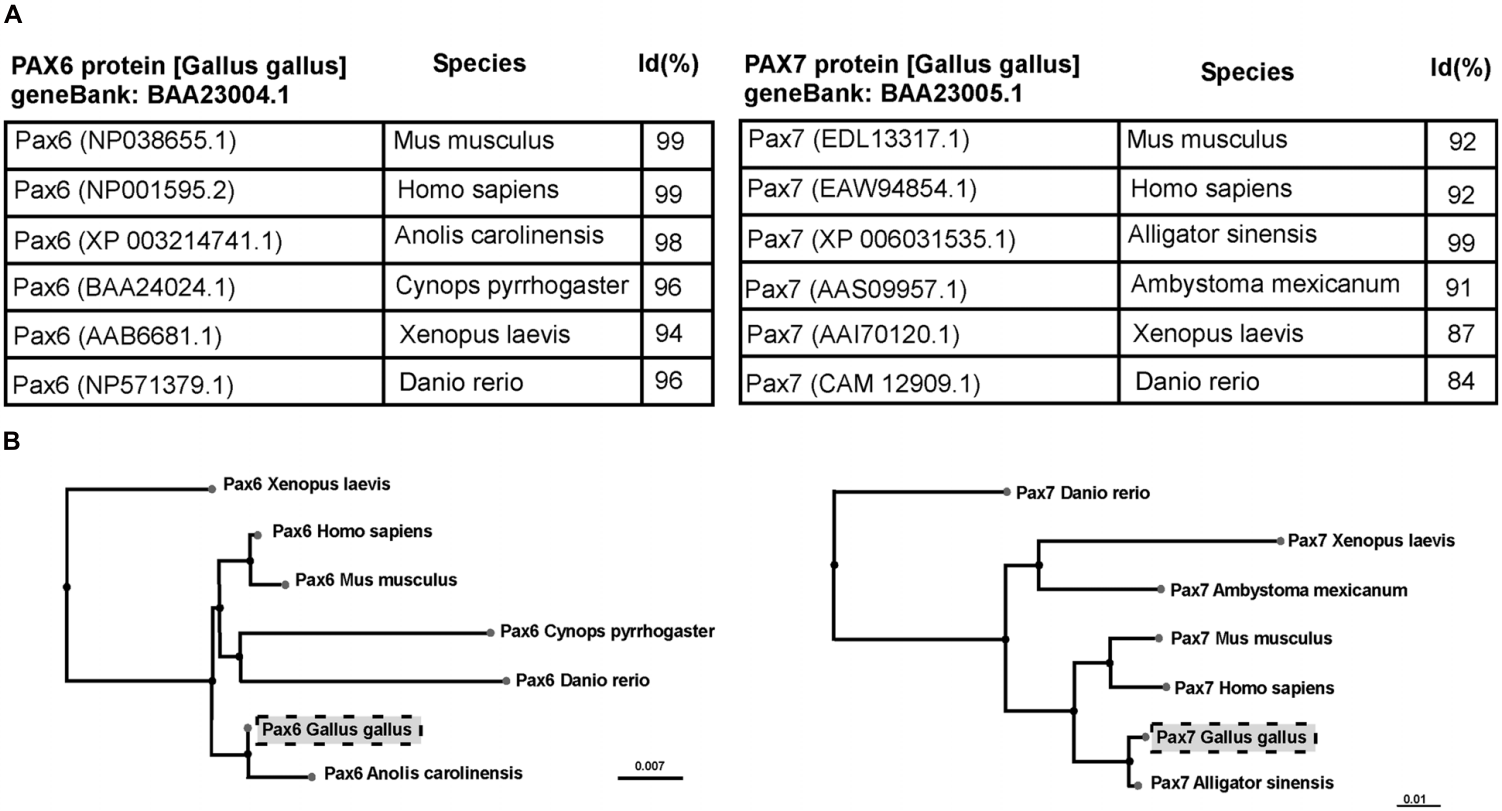
**Table (A) and cladogram (B) of the percentage of homologies (Id%) between species, analyzing the sequence of the Pax6 and Pax7 genes.** Sequences were identified following BLAST (Basic Local Alignment Search Tool) that computes a pairwise alignment between a query and the database sequences searched. Thus, this tool allows to analyze multiple alignments and obtain a sequence tree presentation based on the alignment of database sequences to the query.

The antigen sequence (**Table 1**) has been analyzed by BLAST, which finds regions of local similarity between sequences calculating the statistical significance of matches (http://blast.ncbi.nlm.nih.gov). The sequences analyzed were compared with those published of higher scoring and reliability. In addition, using the BLAST pair-wise alignment a tree view was constructed, using the fast minimum evolution algorithm that calculates the distance between the sequences selected and the evolutionary relationships (**Figure 1**).

**Table 1:** Primary antibodies.

Name	Immunogen	Commercial supplier	MW (kDa)	Dilution
PAX6	*Escherichia coli* derived recombinant chick PAX6. aa 1-223 of the chick Pax6	Monoclonal mouse anti-Pax6; Developmental Studies Hybridoma Bank, Iowa City, IA; Cat. No. PAX6	46	1:250
PAX6	Peptide sequence: QVPGSEPDMS-QYWPRLQ of the C-terminus of the mouse PAX6 protein	Polyclonal rabbit anti-Pax6; Covance, CA; Cat. No. PBR-278	46	1:300
PAX7	*E. coli-*derived recombinant chick PAX7. aa 352–523 of the chick Pax7	Monoclonal mouse anti-Pax7; Developmental Studies Hybridoma Bank, Iowa City, IA; Cat. No. PAX7	55	1:500

In all animal models selected in this study the same antibodies were used (mouse anti-Pax6 and mouse anti-Pax7), both widely employed in previous studies. They were the same antibodies selected for the BLAST sequence analysis. However, in the case of the mouse samples, in which these monoclonal antibodies do not work correctly, we have used a rabbit anti-Pax6 serum (Covance). In both cases the results are consistent to those obtained by other authors, and useful in the evolutionary context presented in this study.

### IMMUNOCHEMISTRY FOR Pax6 AND Pax7

An immunohistofluorescence procedure was conducted with the primary antibody on the free-floating sections that, in all cases, was diluted in 5–10% normal serum of the species in which the secondary antibody was raised in PB with 0.1% Triton X-100 (Sigma) and 2% bovine serum albumin (BSA, Sigma). The protocol included two steps, as follows: (1) Incubation for 72 h at 4°C in the dilution of the primary antibody (see **Table 1**): mouse anti-Pax6 (diluted 1:250, monoclonal mouse anti-Pax6; Developmental Studies Hybridoma Bank, Iowa City, IA, USA; Cat. No. Pax6), rabbit anti-Pax6 (diluted 1:300, polyclonal rabbit anti-Pax6; Covance, CA, USA; catalog No. PBR-278), or mouse anti-Pax7 (diluted 1:500, monoclonal mouse anti-Pax7; Developmental Studies Hybridoma; catalog No. PAX7), and (2) according to the species in which the primary antibody was raised, the second incubations were conducted with the appropriately labeled secondary antibody diluted 1:500 for 90 min at room temperature: Alexa 594-conjugated goat anti-rabbit (red fluorescence; Molecular Probes, Eugene, OR, USA; catalog reference: A11037), Alexa 488-conjugated goat anti-mouse (green fluorescence; Molecular Probes; catalog reference: A21042).

After being rinsed, the sections were mounted on glass slides and coverslipped with Vectashield mounting medium (Vector Laboratories, Burlingame, CA, USA; catalog number: H1000).

The mouse anti-Pax6 and anti-Pax7 antibodies developed by Kawakami et al. (1997) are the most widely used in anatomical studies, independently of the species studied (Ferran et al., 2008, 2009; Morona et al., 2011; Moreno et al., 2012b; Bandín et al., 2013, 2014; Domínguez et al., 2013, 2014; Joven et al., 2013a,b). In order to develop the antibodies against Pax6 and Pax7, Kawakami et al. (1997) first determined the complete nucleotide sequence of the full-length cDNA encoding for Pax6 and Pax7. The antigen used in the case of Pax6 included the paired domain, whereas in the case of Pax7 it was not in the paired domain or in the homeodomain, but closer to the C-terminal region (see Figure 1A in Kawakami et al., 1997). The amino acid sequences of the chick Pax6 and Pax7 are strikingly conserved across the vertebrates for which sequence data are available (see **Figure 1**), with more than 96% sequence similitude. Additionally, the epitopes seem to be also very conserved, given the high quality of results obtained with these antibodies.

### IMAGING

The sections were analyzed with an Olympus BX51 microscope equipped for fluorescence with appropriate filter combinations. Selected sections were photographed using a digital camera (Olympus DP72). Photomicrographs were adjusted for contrast and brightness with Adobe PhotoShop CS4 (Adobe Systems, San Jose, CA, USA) and were mounted on plates using Canvas 11 (ACS Systems International, Santa Clara, CA, USA).

## RESULTS

**FIGURE 2:**
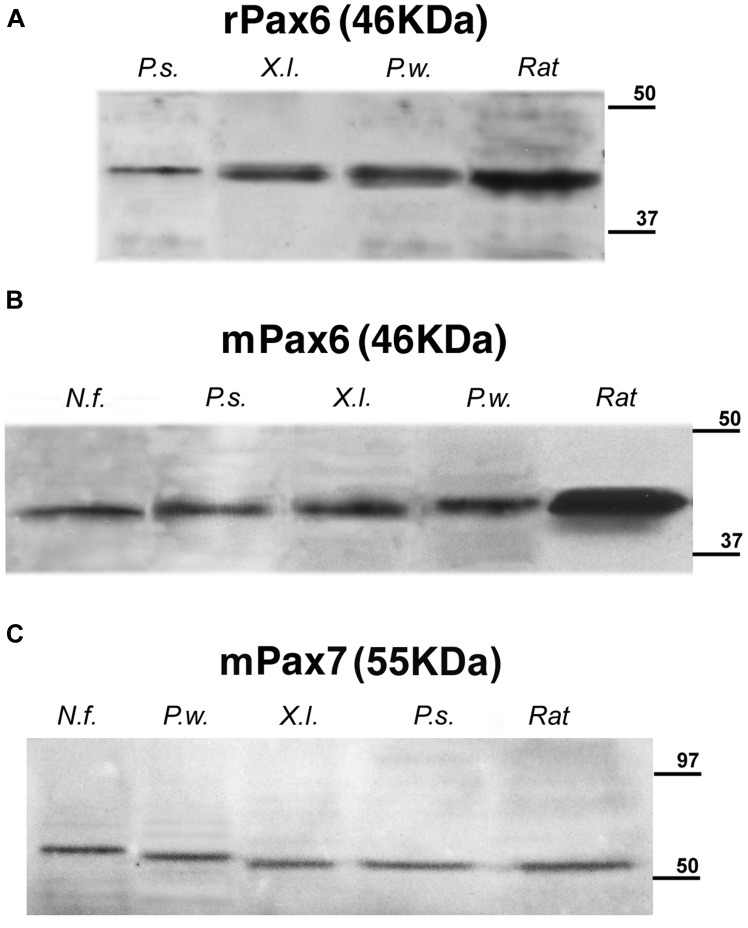
**Identification by Western blots of protein bands recognized by the used antibodies: (A) rabbit anti-Pax6, (B) mouse anti-Pax6, and (C) mouse anti-Pax7.** A single band is seen in each of the lanes corresponding to the *Neoceratodus forsteri* (*N.f.*), *Pseudemys scripta* (*P.s.*), *Xenopus laevis* (*X.l*.), and *Pleurodeles waltl* (*P.w.*) brain extracts that are compared with the band stained in each case for rat brain extracts. The expected molecular weight is indicated for each transcription factor, and the molecular weight standard is represented at right in each photograph.

The specificity of the antibodies had previously been tested in most of the species used in this comparative analysis (Ferran et al., 2009; Moreno et al., 2010; Morona et al., 2011; Domínguez et al., 2013, 2014; Joven et al., 2013a,b). Moreover, the immunoblotting conducted with brain extracts of all the species used showed that the Pax6 and Pax7 antibodies labeled a single band at comparable molecular weight across species (**Figure 2**).

**FIGURE 3:**
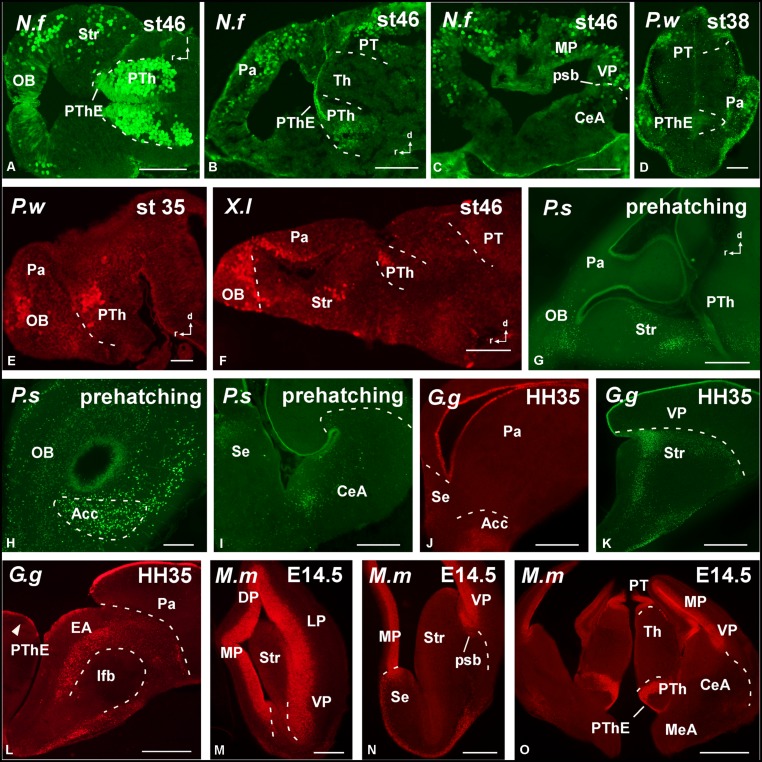
**Photomicrographs of horizontal (A), sagittal (B,E–G), and transverse (C,D,H–O) sections at prosencephalic levels that illustrate rostrally the localization of Pax6-immunoreactive cells in the olfactory bulb of *Neoceratodus forsteri* (A), *Pleurodeles waltl* (E), *Xenopus laevis* (F), and *Pseudemys scripta* (G,H).** In the pallial region, the subventricular zone of anamniotes **(B–F)** and the ventricular zone of amniotes **(I–O)** show Pax6 expression. In the striatal region, Pax6-ir cells are distributed in the striatum proper **(A,F,G,K,M,N)**, the nucleus accumbens **(H,J)**, and in the amygdaloid striatal component of anamniotes and amniotes **(C,I,L,O)**. The prethalamic eminence showed Pax6-ir cells in the ventricular zone of all the species analyzed **(D,O)**. For abbreviations, see list. Scale bars = 200 μm **(A–C,F–O)**, 100 μm **(D,E)**.

**FIGURE 4:**
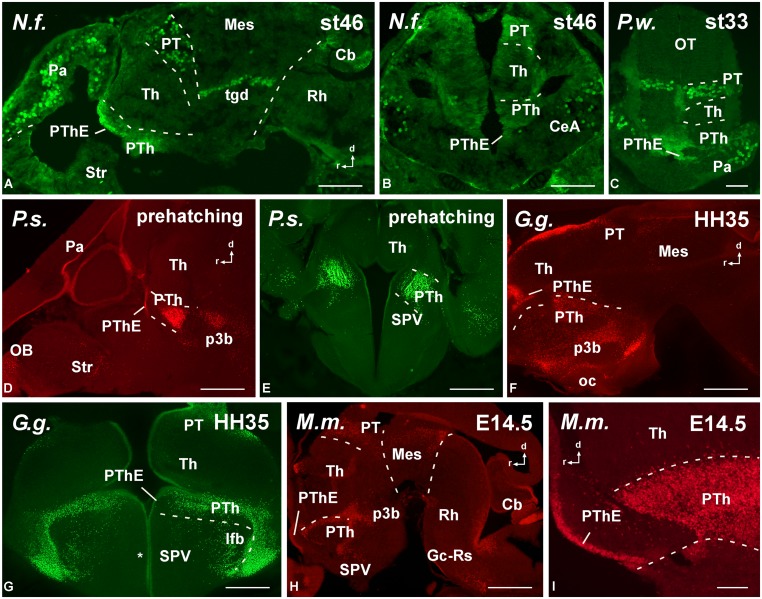
**Photomicrographs of sagittal (A,D,F,H,I) and transverse (B,C,E,G) sections at diencephalic levels that illustrate the localization of Pax6-immunoreactive cells in the ventricular zone of the prethalamic eminence in anamniotes **(A–C)** and amniotes **(D–I)** and the subventricular and mantle zone found in the prethalamus of *Neoceratodus forsteri* (A), *Pleurodeles waltl* (C), *Pseudemys scripta***(D,E)**, *Gallus gallus***(G)**, and *Mus musculus* (H,I).** Asterisk in **G** indicates Pax6 labeling in the SPV ventricular zone. For abbreviations, see list. Scale bars = 200 μm **(A,B,D–H)**, 100 μm **(C,I)**.

**FIGURE 5:**
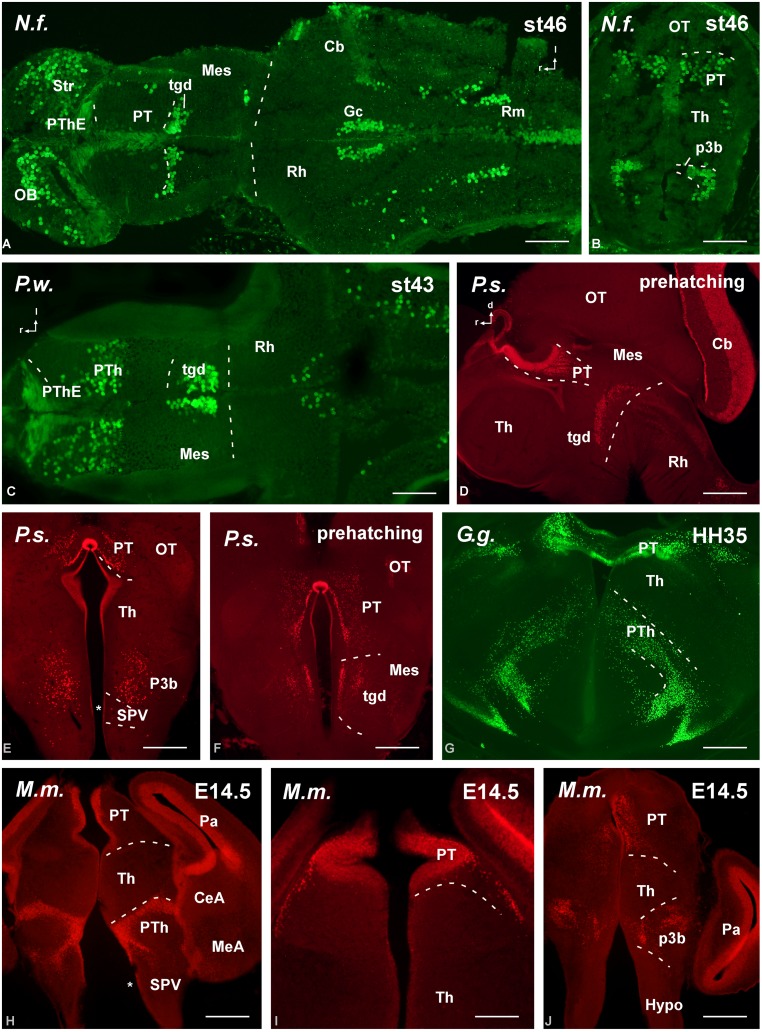
**Photomicrographs of horizontal (A,C), transverse (B,E–J) and sagittal (D) sections at diencephalic levels that illustrate the localization of Pax6-immunoreactive cells in the pretectum of anamniotes (A–C) and amniotes (D,F–I), and in the basal portion of p3 (B,E,H–J).** The ventricular zone of the supraoptoparaventricular region of the alar hypothalamus showed Pax6-ir cells in amniotes (asterisk in **E,H**). For abbreviations, see list. Scale bars = 200 μm **(A–D)**, 100 μm **(E–J)**.

**FIGURE 6:**
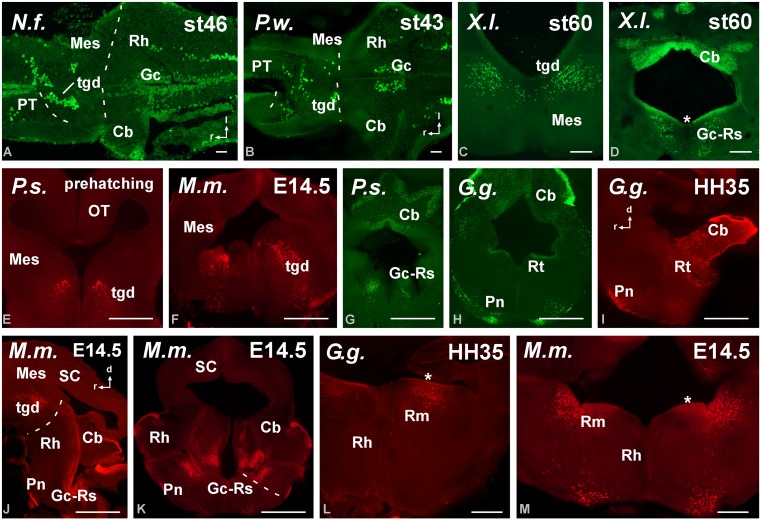
**Photomicrographs of horizontal (A,B), transverse (C–H,K–M) and sagittal (I,J) sections at mesencephalic and rostral rhombencephalic levels that illustrate the localization of Pax6-immunoreactive cells in the dorsal mesencephalic tegmentum in anamniotes (A–C) and amniotes (E,F).** The cerebellum showed Pax6-ir cells in all the models analyzed **(A,G,H,J)**. In the rostral rhombencephalon the griseum centrale **(D,G,J,K)** and the reticular nuclei **(H,L,M)** expressed Pax6 in amniotes and anamniotes, whereas in the pontine nuclei Pax6 expression was found in amniotes **(H–K)**. Asterisks in **D, L,** and **M** indicate labeling in the rostral rhombencephalon. For abbreviations, see list. Scale bars = 200 μm **(A,B,E–M)**, 100 μm **(C,D)**.

**FIGURE 7:**
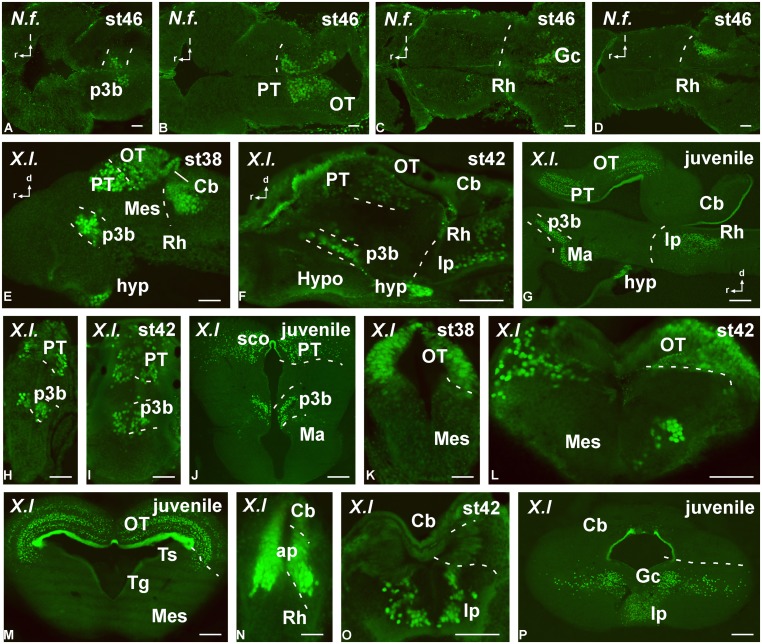
**Photomicrographs of horizontal (A–D), transverse (H–P) and sagittal (E–G) sections that serve to illustrate the localization of Pax7-immunoreactive cells in the developing brain of the anamniotes *Neoceratodus forsteri* (A–D) and *Xenopus laevis* (E–P).** The conspicuous labeling in the diencephalic p3 region is illustrated at different stages **(A,E,F–J)**. Pax7 cells are also illustrated in the pretectum **(B,E,G–J)** and tectum **(B,E,G,K–M)**. Caudally in the brainstem, Pax7 cells are also found in the rhombencephalon **(D–G,N–P)**. For abbreviations, see list. Scale bars = 200 μm **(A–D)**, 50 μm **(E,H,K,N)**, 100 μm **(F,G,I,J,L,M,O,P)**.

**FIGURE 8:**
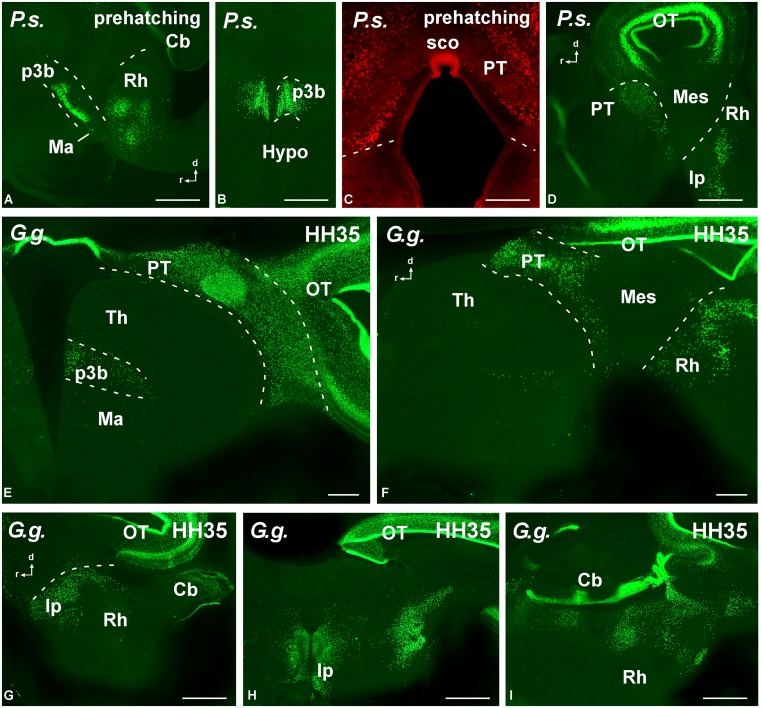
**Photomicrographs of transverse (B,C,E,H,I) and sagittal **(A,D,F,G)** sections that serve to illustrate the localization of Pax7-immunoreactive cells in the brain of the amniotes *Pseudemys scripta* (*P.s.*) just at prehatching stages **(A–D)**, *Gallus gallus* (*G. g.*) at stage HH35 (E–I).** The labeled cells were mainly located in the diencephalic p3 region **(A,B,E)**, the pretectum **(C–F)**, the tectum **(D–H)**, and the rostral rhombencephalon **(A,D,F–I)**. For abbreviations, see list. Scale bars = 200 μm **(A,B,D)**, 100 μm **(C,E–I)**.

The specificity of the antibodies was determined by Western blot (**Figure 2**), and the comparative analysis of the expression patterns for Pax6 (**Figures 3–6**) and Pax7 (**Figures 7** and **8**) was carried out by means of immunofluorescence. We have used developing stages and juveniles of representative species of several vertebrate groups including the lungfish *N. forsteri* (anamniote most closely related to tetrapods), the anuran *X. laevis* and the urodele *Pleurodeles waltl* (amphibians that are the only anamniote tetrapods), and among the amniotes, the reptile *Pseudemys scripta*, the avian *G. gallus*, and the mammalian *M. musculus*. In the following sections, the regional expression patterns observed for Pax6 and Pax7 will be described, indicating for each distinct location whether it was constantly observed across species (conserved feature) or it was a situation exclusive of a particular group. The patterns of labeling for Pax6 and Pax7 are described from rostral to caudal levels and attending to the main subdivisions of the brain. The results were analyzed primarily within the context of recently proposed subdivisions of the telencephalon and the neuromeric organization of the brain, following the current model validated for most vertebrates (*prosencephalon*: Puelles and Rubenstein, 1993, 2003; *midbrain*: Díaz et al., 2000; *rhombencephalon*: Gilland and Baker, 1993; Marín and Puelles, 1995; Cambronero and Puelles, 2000; Aroca and Puelles, 2005; Straka et al., 2006).

### PATTERN OF Pax6 EXPRESSION

#### Forebrain

The primary prosencephalic vesicle gives rise through development to the diencephalon (caudally) and the secondary prosencephalon (rostrally), the latter formed by the telencephalon and the hypothalamus, and all these regions constitute the forebrain (see Puelles and Rubenstein, 2003). Pax6 cells were detected in the rostral parts of the forebrain, mainly in the olfactory bulbs and ventral (subpallial) and dorsal (pallial) regions of the telencephalic hemispheres. Within the olfactory bulbs, Pax6 cells were found in the ventricular and subventricular zones of the embryonic brain of the anamniotes (lungfish and amphibians; **Figures 3A,E,F** and **5A**) that later in development populate the internal granule cell layer, forming a compact cell population, with scattered cells extended peripherally around the glomeruli that define the glomerular layer of the bulb. Similar Pax6 cell populations were observed in amniotes up to juvenile stages (**Figures 3G,H**).

A shared observation in all the species studied was the Pax6 expression in the subpallium, namely in the basal ganglia including the rostral nucleus accumbens (Acc; **Figures 3H,J**) and the more caudodorsal striatum (Str; **Figures 3A,F,M**). In these locations, the ventricular zone was devoid of expression, with the exception of the dorsal most part of the striatum. In amphibians the nucleus accumbens shows intense Pax6 expression (Bandín et al., 2013, 2014; Joven et al., 2013a,b) and in different fish groups the nucleus accumbens was tentatively identified by the presence of Pax6-immunoreactive cells in equivalent areas of the ventromedial telencephalic hemisphere (González and Northcutt, 2009; González et al., 2014). In chicken and mouse it appears that the Pax6-rich dorsal striatal domain also contributes some cells for other ventral areas in the subpallium, including the accumbens shell and the olfactory tubercle (striatal and pallidal parts; Abellán and Medina, 2009). Thus the implication of Pax6 in the accumbens specification seems a very conserved feature in the evolution of vertebrates, at least in a portion of the nucleus.

Pax6 expression was also conserved in medial septal territories from rostral to caudal levels (**Figure 3J**). A striking difference was noted in the pallial Pax6 expression between anamniotes and amniotes because Pax6 cells extended in the mantle zone of the pallium of anamniotes (**Figures 3B,C,E,F**) and, in contrast, Pax6 expression was restricted to the pallial ventricular zone of amniotes (**Figures 3G,J,K,M**). Of note, in all species analyzed the Pax6 expression marked the pallio-subpallial boundary (psb), the distribution of the labeled cells extended into the subpallium (**Figures 3C,N**).

Another constant feature was the intense Pax6 expression observed in the striatal part of the amygdala, in the caudal pole of the telencephalon (**Figures 3C,G,I,L,O**). In addition, more moderate Pax6 expression was commonly found in the medial amygdala, i.e. the part of the amygdaloid complex that receives the bulk of the projection from the accessory olfactory bulb, therefore considered the vomeronasal amygdala (**Figure 3O**).

Distinct pattern of Pax6 cell distribution was observed in the diencephalon. Three segments form the diencephalon, which area named prosomeres 1–3 (p1–p3, from caudal to rostral). It is worth mentioning that in all species these three segments are bent due to the cephalic flexure, so that in conventional “transverse” sections they are observed one at the top of the other, with p1 “dorsal” to p2, and p2 “dorsal” to p3 (see **Figure 4**). The caudal p1 contains in its dorsal part (alar part) the pretectal region, whereas p2 contains the thalamus (former dorsal thalamus) and p3 the prethalamus (PTh; former ventral thalamus). These three prosomeres possess smaller basal (tegmental) regions that are rostrally continuous with the basal hypothalamus. Starting from rostral levels, Pax6 cells were strikingly abundant in the dorsal part of p3, which is currently named “prethalamic eminence.” In early embryos, the weak Pax6 expression was found in the ventricular zone (**Figures 3L,M,O**) that was more conspicuous later in development (**Figures 4A–D,F,H,I**). In addition, in all animals analyzed Pax6 expression was detected in a pattern highly conserved in the subventricular zone of the PTh (see **Figure 4**).

In striking contrast with the abundant Pax6 cell population found in p3 (and also in p1), the second diencephalic prosomere, p2, was virtually devoid of labeling. Only the pineal organ showed Pax6 staining. Actually, the lack of labeling in p2 served to identify, in many cases, the three distinct diencephalic prosomeres.

The roof plate of p1, which contains the subcommissural organ, was intensely labeled for Pax6. The alar derivatives of p1 form the complex pretectal region, which both in anamniotes (**Figures 3D,F**, **4C** and **5A,B**) and amniotes (**Figures 5D–I**) showed ventricular and subventricular Pax6 expression throughout development. In late development, the Pax6 expression was found in rows of cells (**Figures 5F–I**) leaving the ventricular zone and invading distinct nuclei that form the complex pretectal region fully characterized in *Xenopus*, chicken, and mouse (Ferran et al., 2008, 2009; Morona et al., 2011). Pax6 cells were particularly abundant rostrally in p1, extending from the ventricular zone to the most superficial zones, reaching the boundary with the mesencephalic tectum.

In the basal portion of the diencephalon Pax6 was also detected in the animal models studied (**Figure 5**). Thus, in the basal portion of p3 a population of scattered cells expressing Pax6 was detected (**Figures 4D,F** and **5B,E,G,H,J**) that occasionally reached adjacent hypothalamic territories. Actually, a striking difference between anamniotes and amniotes was noted in the hypothalamus because in amniotes Pax6 expression was found in the ventricular zone of the alar part of the hypothalamus (see asterisks in **Figures 4G** and **5E,H**), namely in the supraoptoparaventricular region (SPV), whereas in the anamniotes studied only the urodele amphibian *Pleurodeles* showed transitory hypothalamic Pax6 expression during the development (Joven et al., 2013a,b).

#### Brainstem

The localization of Pax6 cells in the caudal domain of the pretectum and the lack of Pax6 expression in the dorsal midbrain (mesencephalon) highlighted the diencephalo–mesencephalic boundary. In contrast, in all species studied a conspicuous Pax6 cell population extended along the dorsal part of the mesencephalic tegmentum, just below the alar/basal boundary (**Figures 5A,C,D,F** and **6A–C,E,F**). These cells formed a small group rostrally, at the level of the rostral pole of the oculomotor nucleus, whereas caudally they were more widely distributed into cell bands (**Figures 6C,E**). This band of Pax6 cells ended abruptly at the boundary with the isthmus (r0; e.g., **Figure 4A**).

Pax6 expression was practically absent from the isthmus (segment r0), which is severely curved in all species, given the obliquity of the isthmomesencephalic boundary. As a derivative of the alar part of segment r1, the cerebellum showed Pax6 expression in the granule cells of the cerebellar plate and auriculae in all species (**Figures 5D** and **6A,B,D,G–K**).

Also in all species studied, Pax6 expressing cells were detected in the rostral rhombencephalon (see asterisks in **Figures 6D,L,M**), and even at early developmental stages, at this rhombencephalic levels, the griseum centrale and the reticular nuclei expressed Pax6 (**Figures 6D,G–K**). In amniotes, Pax6 expression was also found in the pontine nuclei (**Figures 6H–K**). Caudally, Pax6 labeling was observed in the ventricular zone of the basal plate throughout the rhombencephalon. Close to the obex, once the central canal is formed, distinct Pax6 labeling of the ventral ventricular zone was observed and Pax6 cells were labeled detached from the ventricular zone into the ventrolateral region of the somatomotor spinal neurons. Noteworthy, in the spinal cord the Pax6 cell populations persisted in the ventral spinal cord in the juveniles but the labeling of the ventricular zone disappeared.

### PATTERN OF Pax7 EXPRESSION

#### Forebrain

Regarding the presence of Pax7 cells in the rostral prosencephalon, only a conspicuous labeling was identified in the paraphysis, mainly in anamniotes, in relation to the choroid plexus that extended between the telencephalic hemispheres. In addition, a few Pax7 cells were located in the caudobasal hypothalamus within the mammillary region, in close relation to the diencephalic cell population in p3 (see below). Of note, conspicuous Pax7 cells formed a dense population in the intermediate lobe of the hypophysis (**Figures 7E–G**).

Actually, among the most conspicuous Pax7 cell populations in the brain observed in all species was the group of neurons labeled in the basal part of p3 (**Figures 7** and **8**). These cells formed a band of packed neurons in the rostral part of the basal p3, close to the boundary with the hypothalamus, which is topologically rostral, and were observed from early developmental stages to the adult in anamniotes (**Figures 7A,E–J**) and also in amniotes (**Figures 8A,B,E**). Of note, scattered Pax7 cells located more ventral in p3 extended along development into regions of the adjacent basal hypothalamus, in particular within the mammillary region, and this was better observed in anamniotes (**Figures 7G,J** and **8A**).

As for the case of Pax6, the prosomere p2 lacked Pax7 expression, although the membranous roof plate between the two dorsal habenular components was intensely labeled for Pax7 particularly in *Xenopus*.

A large population of Pax7 cells was consistently localized in the dorsal part of p1 of all species studied (**Figures 7B,E–J** and **8C–F**). The subcommissural organ in the roof plate was Pax7 positive (**Figures 7J** and **8C**). In the pretectal region, Pax7 cells were abundant throughout the intermediate region, named juxtacommissural region (after Ferran et al., 2007, 2009). Also, abundant Pax7 cells occupied the caudal region of the dorsal p1, close to the diencephalo–mesencephalic boundary (**Figures 7E,G,J** and **8D,E**).

#### Brainstem

The most conspicuous labeling in the mesencephalon was found in the optic tectum (superior colliculus in mammals) where Pax7 cells were abundant from early stages. During the embryonic development, the mesencephalic neuroepithelium expressed Pax7 in broad domains, in both the rostral and caudal mesencephalic poles. As development proceeded, the Pax7 expression showed a caudo-rostral gradient in which the expression is gradually weaker toward the rostral pole. These gradients were observed in all the species studied. Progressively, the distinct cell layering in the tectum of all species was observed, including intensely labeled cells in the ventricular zone that persisted in the juveniles (**Figures 7E,K** and **8D**). The extent of this patent labeling ended caudally at the border between the optic tectum and the torus semicircularis (inferior colliculus in mammals), although scattered cells were seen in this caudal part (**Figures 7L,M**). The mesencephalic tegmentum lacked Pax7 expression in all species at all developmental stages.

Pax7 cells were particularly abundant in the rostral rhombencephalon in r1, which is a large rhombomere that extends from the caudal pole of the trochlear nucleus in r0 to the rostral pole of the trigeminal motor nucleus in r2. Pax7 cells were early labeled in the ventricular zone of the alar plate (including the ventricular zone of the cerebellum; **Figure 7N**). Subsequently during development, many cells appeared to migrate tangentially into the adjacent basal plate (**Figure 7O**) to reach the interpeduncular nucleus (**Figure 7P**). This situation is conserved through amniotes (**Figures 8D,H**), where Pax7 positive cells of the alar plate migrate into the basal plate during development to finally be a subpopulation of the interpeduncular complex (Lorente-Cánovas et al., 2012).

Along the rhombencephalon, Pax7 labeling was found in the ventricular zone of the alar plate in all species, being more intense in the ventral part of the alar plate than in the dorsal part. Separate Pax7 cells from the ventricular zone were scarce, mainly the level of the nucleus of the solitary tract.

Close to the obex distinct Pax7 labeling was observed in the ventricular zone of the dorsal part of the central canal. In addition, in the dorsal region a compact group of Pax7 cells migrated from the ventricular zone was intensely labeled at the obex region and caudally in the spinal cord.

## DISCUSSION

All studies in comparative biology depend upon robust phylogenetic frameworks. Besides the corroboration of many of the traditional morphology-based phylogenetic relationships, new molecular data sets have also been particularly helpful in discerning among competing hypothesis. Thus, a comparative study in which main animal groups are analyzed together seems very interesting and can be a starting point for establishing basic neuroanatomical relationships.

### CHOICE OF SPECIES TO STUDY SHARED FEATURES IN TETRAPODS

Sarcopterygians form a monophyletic group including living species of lobe-finned fishes and tetrapods (Hallstrom and Janke, 2009; Chen et al., 2012) that diverged from the ray-finned fishes about 450 million years ago (mya). Most recent data obtained through phylogenomic analysis concluded that the lungfishes are the closest living relatives of tetrapods (Brinkmann et al., 2004; Chen et al., 2012; Amemiya et al., 2013) and the Australian species *N. forsteri* seems to have retained most of the primitive traits of ancient lobe-finned fishes (Kemp, 1987). Therefore, it seems reasonable that many features observed in this species may resemble closely those of ancestral tetrapods, which gives us a unique window into the evolution of the CNS in tetrapods, from an aquatic fish ancestor.

Amphibians constitute the first lineage of tetrapods that likely appeared in the Permian (reviewed in Meyer and Zardoya, 2003), and the separation of the three orders of modern amphibians (Anura, Caudata, and Gymnophiona) probably occurred almost immediately (in evolutionary time) after the “jump to land” of lobe-finned fishes (360 mya; San Mauro et al., 2005). They are the only group of anamniote tetrapods and their study is very interesting since essentially they constitute a transition model in the evolution of vertebrates.

The living amniotes have traditionally been divided into three groups based on the fenestration of their skulls. The anapsids (without holes in their skulls) represented by the turtles, the synapsids (with one hole) composed by mammals, and the diapsids (with two holes) represented by the rest of groups. The molecular phylogeny of tetrapods is presently among the best documented (Meyer and Zardoya, 2003). Historically, the turtles were considered as the only living survivor of anapsid reptiles and therefore as the sister group of all living amniotes (Gaffney, 1980). More recent phylogenetic analysis supported the phylogenetic position of turtles closer to reptiles (Meyer and Zardoya, 2003; Fong et al., 2012), but some authors support that turtles are the sister group of archosaura (birds and crocodiles; Werneburg and Sánchez-Villagra, 2009; Chiari et al., 2012; Crawford et al., 2012). What seems clear is that, in general terms, morphologists and paleontologists now believe that crocodiles are the closest living relatives to birds and both groups are the only surviving lineage of the Archosaura (Gaffney, 1980; reviewed in Meyer and Zardoya, 2003). Therefore, the analysis of both turtles and birds provides a representation of each group.

In this context, the species selected for our study clearly serve to gain insight into the evolutionary traits of the Pax6 and Pax7 expression patterns in the brain of sarcopterygians, from lungfishes through mammals.

### CONSERVED EXPRESSION PATTERNS

The concept of novelty in evolutionary biology affects multiple levels of biological organization, from behavioral and morphological changes to changes at the molecular level. Thus, to identify the new features it is necessary to analyze the phylogenetic history in terms of similarity and shared developmental and genetic pathways or networks. Novel phenotypes can be generated through both neofunctionalization and gene rearrangements. Therefore, assigning phenotypic or genotypic “novelties” is contingent on the level of biological organization addressed (Hall and Kerney, 2012).

Additional expression domains for Pax genes arose in vertebrates subsequent to gene duplication and the evolution of new structures. Thus, the comparison of these expression patterns in this case seems of interest because specific evolutionary differences can be interpreted and discussed in evolutionary terms in the context of particular adaptations. Moreover, the definition of different progenitor regions in the nervous system was first based, largely, on anatomical landmarks, such as sulci and bulges. Unfortunately, despite the convenience of anatomical references, morphological boundaries do not always coincide with molecular limits, they are often misleading because they may change in position over time, and, most important in evolutionary perspective, they are not always comparable among species. This is one of the reasons for the comprehensive gene expression analysis conducted in recent years in different species, which led to establish homology relationships in terms of genetic specification of neural territories. However, in some cases expression territories of developmental genes can also be misleading, either because their expression limits do not correspond to morphogenetic entities, or because paralogous exchange expression territories depending on the species.

In this context, studies on the ontogeny of the brain in many different species are abundant, especially in mammals, but in many respects they provide contradictory data. This is generally due to the fact that most studies are based on the now outdated columnar model of Herrick (1910), which is inconsistent with gene expression patterns and essentially offered conjectures about the areas where cell populations arise. In the meantime, the prosomeric model has been postulated as an alternative conceptual scenario (considering a different longitudinal axis), consistent with the gene expression evidence (for review see Puelles et al., 2012a). This model has allowed very precise definition of diverse progenitor domains, each characterized by a differential molecular code.

Pax expression has been reported in distinct cell masses in diverse regions of the CNS in some representatives of all major vertebrate classes. Although most data are restricted to specific regions during development, the patterns of expression of each subpopulation described are largely comparable across species. In particular, the Pax6/7 genes are the first to appear in the developing CNS. With the exception of Pax6, which excludes the mesencephalic roof from its expression domains, these genes are present in the entire developing neural tube. During development, Pax6 is abundantly expressed in the forebrain (telencephalon and diencephalon), whereas Pax7 withdraws from the telencephalon having its rostral limit in the diencephalon. Both transcription factors have been involved in brain circuitry formation. Thus, Pax6 expression acts in determining graded topography in the retina (Ziman et al., 2001) or the cortex (Stoykova et al., 1996) while Pax7 is fundamental for optic tectum (superior colliculus) development (Thomas et al., 2004).

#### Comparative expression patterns in the forebrain

***Olfactory bulbs.*** The presence of Pax6 in the olfactory bulbs is a conserved feature in vertebrates and has been reported from lampreys through mammals, including humans. During mouse development Pax6 is essential for the formation of the olfactory placode, olfactory bulb, and olfactory cortex (Nomura et al., 2007). Furthermore, it is required for the differentiation of granule and periglomerular cells in the postnatal and adult olfactory bulb (Hack et al., 2005; Kohwi et al., 2005). Actually, Pax6 is required for the differentiation and/or maintenance of specific subtypes of interneurons in the adult olfactory bulbs (Haba et al., 2009) and the implication of Pax6 in neurogenesis and periglomerular dopaminergic cells fate specification in the olfactory bulbs has recently been demonstrated (Agoston et al., 2014). A similar situation might be present in most vertebrates where Pax6 is found in dopaminergic cells during development and in the adult (Wullimann and Rink, 2002; Hack et al., 2005; Kohwi et al., 2005; Vergaño-Vera et al., 2006; de Chevigny et al., 2012; Quintana-Urzainqui et al., 2012a; Bandín et al., 2013; Joven et al., 2013b).

***Pallium.*** The Pax6 expression in the pallium of vertebrates is a conserved feature in all the models analyzed. Pax6 was previously considered a general marker of the ventricular zone of the pallium during development (Puelles et al., 2000) but neurons expressing Pax6 in the subventricular zone of adult mice were reported in a recent immunohistochemical study (Duan et al., 2012), in line with results obtained in a number of nonmammalian vertebrates (Wullimann and Rink, 2001; Moreno et al., 2008; Abellán and Medina, 2009; Moreno et al., 2010; Bandín et al., 2013; Joven et al., 2013a,b). The pallium seems to lose Pax6 expression in the subventricular zone after development, with the exception of the most ventral portion corresponding to the ventral pallium, in the psb zone. However, fragmentary data from elasmobranches (*Scyliorhinus canicula*; Ferreiro-Galve et al., 2008) and lungfishes (*Protopterus dolloi*: González and Northcutt, 2009; *N. forsteri*: present results) suggest that abundant Pax6 expression remains in pallial cells after development.

In this context, the psb was originally defined in various vertebrates (mouse, chick, turtle, and frog) based on its Pax6 expression and the lack of Emx1 expression, found in all other pallial regions (Smith-Fernández et al., 1998). That constituted the origin of the identification of the ventral pallium (Puelles et al., 2000), currently recognized in most vertebrate groups (Puelles et al., 2000; Brox et al., 2004; Medina et al., 2004; Moreno and González, 2004; González and Northcutt, 2009). In mammals, the psb is a complex region that might influence cell migration between the subpallium and pallium, and controls the migration of pallial cells ventrally to the striatum (Fishell et al., 1993; Chapouton et al., 1999; Marín and Rubenstein, 2003; Carney et al., 2006). In addition, in all the species analyzed Pax6 cells from the psb appear to migrate to specific emerging amygdaloid nuclei and other basal telencephalic structures (Carney et al., 2006; Ferreiro-Galve et al., 2008; Moreno et al., 2008; Rodríguez-Moldes, 2009; Quintana-Urzainqui et al., 2012b; Bandín et al., 2013, 2014; Joven et al., 2013a,b; present results). Therefore, the analysis of this psb zone in vertebrates with different pallial and subpallial features seems of great interest from a comparative perspective, because differences in the organization of the psb could be essential in the evolution of pallial differences.

***Basal ganglia.*** The basal ganglia share a common pattern of organization in vertebrates, including the presence of the striatal and pallidal components (for review see Reiner, 2010; Stephenson-Jones et al., 2012; González et al., 2014). In amniotes, Pax6 is expressed in the most dorsal domain: in mammals named as lateral ganglionic eminence 1 and 2 (LGE1 and LGE2; Flames et al., 2007; Pauly et al., 2014), and named the dorsal striatal domain in birds (Abellán and Medina, 2009) or the dorsal striatum in reptiles (Moreno et al., 2010).

Adult mice appear to lack Pax6 cells in the striatum (Stoykova and Gruss, 1994; Duan et al., 2012), whereas in chickens Pax6 neurons persist at the lateral edge of the ventral striatum, forming a distinct cell mass postnatally (Puelles et al., 2000). In adult turtles, Pax6 expression was observed in the striatum in migrated cells located near the pial surface (Moreno et al., 2010). In addition, in amniotes, the primordium of nucleus accumbens is immediately caudal to the olfactory bulb and expresses Dlx2 and Pax6 in cells entering the mantle zone radially (Puelles et al., 2000; Moreno et al., 2010). Also in amphibians the nucleus accumbens shows intense Pax6 expression (Bandín et al., 2013, 2014; Joven et al., 2013a,b). In different fish groups, the nucleus accumbens was tentatively identified by the presence of Pax6-immunoreactive cells in equivalent areas of the ventromedial telencephalic hemisphere (González and Northcutt, 2009; González et al., 2014). Thus the implication of Pax6 in the accumbens specification seems a very conserved feature in the evolution of vertebrates.

***Septum.*** Several developmental studies have demonstrated that the septum in amniotes is essentially a subpallial derivative, but a contribution from pallial adjacent areas has also been described (Puelles et al., 2000). In mammals, the septal region closest to the psb is characterized by the expression of Pax6 and the lack of Nkx2.1 transcripts (Flames et al., 2007). In adult mice, moderate Pax6 expression was observed in the lateral septal nucleus, whereas strong signal was detected in the medial septal nucleus and in the horizontal and vertical limbs of diagonal band of Broca (Stoykova and Gruss, 1994; Duan et al., 2012). In the chicken, the rostrodorsal part of septum shows Pax6 expression in the ventricular zone and Tbr-1 in the mantle, and it was interpreted as a putative pallial component of the septum (Puelles et al., 2000). In the turtle *Pseudemys,* the Pax6 expression in the ventricular and mantle zones delineates rostrocaudally a dorsal septal region, included in the GABA-expressing territory and located dorsal to the TH terminal field of the lateral septum (Moreno et al., 2010), generally identified as a striatal septal subdivision. In amphibians, Pax6 expressing cells are mainly present, from rostral to caudal levels, in the most dorsal septal component, called dorsal septum (Moreno et al., 2008; Bandín et al., 2013, 2014; Joven et al., 2013a,b). Also in lungfishes Pax6 cells occupy the dorsal part of the medial septal region (González and Northcutt, 2009; present results). Therefore, the septal Pax6 expression is largely comparable through tetrapods and lungfishes, whereas in actinopterygian fishes identification of the homologous parts of the septal components awaits demonstration, and Pax6 expression can provide highlights to this issue (Ganz et al., 2012; González et al., 2014).

***Extended amygdala.*** The amygdaloid complex has been interpreted as a continuum through the basal forebrain (extended amygdala) including other structures such as the bed nucleus of the stria terminalis (BST; Alheid and Heimer, 1988). In all the models studied the amygdaloid complex includes a striatal territory, usually called central amygdala, rich in Pax6 cells (present results). Previous data in the mouse showed that the central amygdala originates in the LGE subdivision (Puelles et al., 2000; Tole et al., 2005; García-López et al., 2008; Waclaw et al., 2010). More specifically, dorsal LGE-derived neurons expressing Pax6 primarily populate the central amygdala, but a few also reach the lateral bed nucleus of the stria terminalis (Bupesh et al., 2011a). The avian subpallial central extended amygdala, also a Pax6 expressing zone, includes the striatal amygdala and part of the BST (Abellán and Medina, 2008, 2009), as in mammals. Comparatively, in the turtle, a central amygdala is identified as a GABA territory rich in Pax6 cells with dispersed immigrant Nkx2.1 cells (Moreno et al., 2010). Within anamniotes, Pax6 cells are present in the central amygdala of the amphibians *Pleurodeles* and *Xenopus* (Bandín et al., 2013, 2014; Joven et al., 2013b), and in the dogfish a comparable striatal amygdaloid territory expressing Pax6 was described (Quintana-Urzainqui et al., 2012b).

Regarding the Pax6 cells found in the amygdala, observations throughout development in amphibians (Bandín et al., 2013; Joven et al., 2013b) and the dogfish (Quintana-Urzainqui et al., 2012b) have suggested that the amygdala might receive cells from outside the telencephalon, including the hypothalamus and the prethalamic eminence, like in mammals (Tole et al., 2005; Abellán and Medina, 2009; Moreno et al., 2010; Bupesh et al., 2011b). In particular, *in vitro* migration assays for analyzing the origin of the neurons of the medial extended amygdala in mouse embryos demonstrated a minor subpopulation of Pax6-expressing neurons, which does not originate in dorsal LGE but instead may immigrate from the prethalamic eminence (Bupesh et al., 2011b). A contribution of Pax6-expressing cells from the prethalamic eminence is also likely to be present in the chicken extended amygdala (Abellán and Medina, 2009).

In mammals, the lateral BST also includes a subpopulation of Pax6-expressing cells, which derive from either dorsal LGE (Bupesh et al., 2011a). A similar situation was proposed for birds and turtles in which Pax6 cells of striatal origin invade the BST (Abellán and Medina, 2009; Moreno et al., 2010). Interestingly, in adult amphibians Pax6 expression is lacking in the region of the BST (Bandín et al., 2013, 2014; Joven et al., 2013a,b) and is restricted to adjacent striatal zones, what led to suggest that the whole BST has a pallidal origin, expressing Nkx2.1 (Moreno et al., 2012a). Similarly in lungfishes, the region identified as the BST is devoid of Pax6 cells (González and Northcutt, 2009; present results). Therefore, Pax6 expression in the BST seems different between amniotes and anamniotes.

As we have mentioned before, all pallial subdivisions express Pax6 in the ventricular zone in all vertebrates analyzed (Bulfone et al., 1995, 1998; Smith-Fernández et al., 1998; Puelles et al., 2000; present results). Thus, in mammals and likely in vertebrates in general, the pallial progenitor sectors that produce the pallial amygdala also express Pax6 at the ventricular zone (Medina et al., 2004; Tole et al., 2005).

***Hypothalamus.*** The recently updated prosomeric model holds that the hypothalamus is a rostral forebrain entity, ventral to the telencephalon and rostral to the diencephalon, and is subdivided dorsoventrally into alar and basal regions (reviewed in Puelles et al., 2012a). The alar portion includes the suprachiasmatic and the SPV regions, which will give rise to their respective hypothalamic nuclei. The basal hypothalamus includes the tuberal region, which contains among other structures the ventromedial and the arcuate nuclei, and the mammillary region, which includes the subthalamic nucleus (reviewed in Moreno and González, 2011).

At the dorsal boundary with telencephalic subpallium, a narrow strip of cells expressing Nkx2.2 and Pax6 defines the preoptohypothalamic boundary in mammals (Flames et al., 2007; Pauly et al., 2014). Comparable expression pattern in the counterpart boundary region in birds and reptiles has been reported (Bardet et al., 2006; Moreno et al., 2012b). By contrast, in anamniotes this region is indistinguishable on the basis of comparable Pax6/Nkx2.2 expression, as observed in *Xenopus* (Moreno et al., 2008; Domínguez et al., 2011, 2013, 2014; Bandín et al., 2013, 2014), *Pleurodeles* (Joven et al., 2013a,b), lungfishes (Moreno and González, 2011), and lamprey (Murakami et al., 2001).

Within the hypothalamus, Pax6 expression in amniotes has been reported in the ventricular zone of the SPV region (Flames et al., 2007; Abellán and Medina, 2009; Moreno et al., 2010, 2012b), but in the same zone Pax6 expression was not observed in the amphibian *Xenopus* (Moreno et al., 2008; Bandín et al., 2013, 2014; Domínguez et al., 2013, 2014), as was also the case in the lamprey hypothalamus (Murakami et al., 2001). However, during development the urodele amphibian *Pleurodeles* shows transitory Pax6 expression in the SPV region (Joven et al., 2013b). Interestingly, in urodeles Pax6 has been demonstrated to be necessary for the formation of the alar hypothalamic region (Eagleson et al., 2001). A previous study in Xenopus related the change in this Pax6 expression to a variation in the expression of Nkx2.1 in the alar hypothalamus (van den Akker et al., 2008). In the mouse, the absence of Nkx2.1 expression in the alar hypothalamus might correlate with the expression of Pax6 (van den Akker et al., 2008) following opposing roles, as they do in dorsoventral telencephalic patterning where they are primarily expressed in mutually excluding domains (for review see Moreno et al., 2009).

Regarding the Pax7 expression in the hypothalamus, it was described within the Nkx2.1-positive basal hypothalamic progenitors during chicken development (Ohyama et al., 2008), in the mammillary region of the turtle (Moreno et al., 2012b), and in the subthalamic nucleus of mice during development and postnatally (Stoykova and Gruss, 1994). In the case of anamniotes, scattered Pax7 cells have been observed in the mammillary region in amphibians and lungfishes (Bandín et al., 2013, 2014; Joven et al., 2013b; Domínguez et al., 2014; present results). Thus, in all vertebrates that we have analyzed during development and later, Pax7 is expressed in the basal plate of p3 (see below) and in scattered cells in the mammillary region and/or the subthalamic nucleus (present results; see also the Allen Developing Mouse Brain Atlas). This is of special interest since there are discrepancies in the literature about the origin of the subthalamic nucleus in amniotes, currently regarded as a dorsally migrated hypothalamic cells mass, which originated from the retromammillary area, i.e. it belongs to the hypothalamus (Martin et al., 2004; Skidmore et al., 2008; Puelles et al., 2012a). Alternatively, it was considered a derivative of the basal plate of p3, which generates the retromammillary tegmentum and the subthalamic nucleus (García-López et al., 2009). Comparatively, in *Xenopus* and *Pleurodeles*, from very early stages of development the basal plate of p3 is characterized by the ventricular expression of Pax7 and Nkx2.1, and along development scattered cells characterized by this double-expression are progressively located in the mammillary area, likely migrating from the adjacent p3 region (Bandín et al., 2013; Joven et al., 2013b; Domínguez et al., 2014; present results).

***Diencephalon.*** According to the prosomeric model, the diencephalon is subdivided into three segments, prosomeres 1–3 (p1–p3). These contain in their alar regions the PTh plus the prethalamic eminence in the rostral p3, the thalamus plus the habenula or epithalamus in the intermediate p2, and the pretectum in the caudal p1. The smaller basal components form the tegmental region in the diencephalon, extending in the three prosomeres (Puelles and Rubenstein, 2003; reviewed in Puelles et al., 2012b).

In the mouse diencephalon, the alar–basal plate boundary was defined as the ventral extent of alar Pax6 expression (Hauptmann and Gerster, 2000; Mastick and Andrews, 2001; Hauptmann et al., 2002; Ferran et al., 2007, 2008) and, together with Nkx2.2, Pax6 has been implicated in the correct dorsoventral patterning of the diencephalon (Pratt et al., 2000). Within p3 in all vertebrates studied, the prethalamic eminence shows ventricular Pax6 expression while the PTh is filled with Pax6 expressing cells also in the subventricular and mantle zones (Puelles et al., 2000; Wullimann and Rink, 2001, 2002; Bachy et al., 2002; Moreno et al., 2008, 2010, 2012b; Pritz and Ruan, 2009; Duan et al., 2012; Domínguez et al., 2013; present results). In the alligator, the boundary between p3 and the secondary prosencephalon was defined by Pax6 expression, and Pax6 cells were also reported in the basal part of p3 (Pritz and Ruan, 2009). In the turtle *Pseudemys scripta*, scattered Pax6 cells were seen to invade the basal portion of p3 (Moreno et al., 2012b). Comparatively, in *Pleurodeles* (Joven et al., 2013a,b) and *Xenopus* (Bandín et al., 2013, 2014) complementary Pax6/Pax7 expression patterns were observed in the basal part of p3.

The thalamus did not show any Pax6/7 expression after early development in any of the species studied (present results). Actually, the thalamus can be distinguished from the PTh (rostrally) and the pretectum (caudally) by the lack of Pax6/7 expression in the thalamus, in contrast to the alar parts of p3 and p1 (Walther et al., 1991; Stoykova and Gruss, 1994; Duan et al., 2012). Pax6 appears to be important for this boundary formation, as demonstrated in null mutants (Mastick et al., 1997), and early expression of Pax6 in the thalamus needs to be downregulated in order to produce a normal thalamus (Grindley et al., 1997), remaining restricted to the epithalamus (Grindley et al., 1997; Pratt et al., 2000).

In all amniotes and anamniotes studied, Pax7 and Pax6 are expressed in the pretectum (p1; present results), helping in the delineation of its three main subdivisions (for details see: Ferran et al., 2007, 2009; Morona et al., 2011; Bandín et al., 2013, 2014; Joven et al., 2013a,b). Especially, the rostral boundary of the pretectal region is defined molecularly primarily by the expression of Pax6 and Pax7 (Ferran et al., 2007, 2009; Moreno et al., 2008, Morona et al., 2011; present results). Pax6 function is known to define this boundary in mammals by repression of the midbrain centered markers En1 and Pax2 (Matsunaga et al., 2000). Pax6 expression has also been noted in the basal plate of p1 in representatives of all vertebrate groups studied.

#### Comparative expression patterns in the brainstem

The transcription factor Pax6, expressed rostral to the midbrain in the alar diencephalon, contributes jointly with other molecular signals to establish the mes-diencephalic boundary, partly by downregulation of midbrain characteristic markers (reviewed in Puelles et al., 2012). Especially, the absence of Pax6 in the alar midbrain has been used extensively to discriminate between p1 and the mesencephalon. In addition, in the basal plate of all vertebrates analyzed, Pax6 cells form a longitudinal band, located ventral to the midbrain alar–basal boundary (Stoykova and Gruss, 1994; Vitalis et al., 2000; Wullimann and Rink, 2001; Ahsan et al., 2007; Bayly et al., 2007; Pritz and Ruan, 2009; Duan et al., 2012; Bandín et al., 2013, 2014; Joven et al., 2013a,b; present results).

Distinctly, Pax7 is expressed in the optic tectum (superior colliculus) from early embryonic development through the adult in all species studied. In adult chickens, Pax7 was found in neurons located mostly in the outer layers of the optic tectum (Shin et al., 2003). Also in chickens, the crucial role of Pax7 in tectal development was demonstrated because the ectopic Pax7 expression in the diencephalon was proved to induce the formation of an ectopic tectum (Matsunaga et al., 2000). Further, during development Pax7 is involved in establishing tectal polarity (Thomas et al., 2004) and the retino-tectal topography (Thomas et al., 2006). In adult rodents, Pax7 expression is concentrated in neurons located in the retino-recipient laminae (Thomas et al., 2004), and it likely has a role in retinotopic mapping (Thompson et al., 2007).

The hindbrain or rhombencephalon comprises the isthmic segment, frequently named rhombomere 0 (r0), and the rhombomeres 1–11 (r1–11), numbered from rostral to caudal. During embryonic development, the rhombencephalon expresses Pax7 in the alar neuroepithelium and Pax6 is restricted to more ventral domains, in the dorsal part of the basal ventricular zone, with partial overlap with the Pax7 expression zone (present results). In developing amphibians, conspicuous Pax7 cell groups are widely distributed in the large r1, starting at early embryonic stages (Bandín et al., 2013; Joven et al., 2013b), like in birds and mammals (Aroca and Puelles, 2005). Comparatively, it is interesting to note that several neuronal populations were reported (in chick and mouse embryos) to be generated in the r1 alar plate, which migrate ventralwards into the medial basal plate, forming a sizeable part of the interpeduncular nucleus complex, apart other medial tegmental nuclei, along their migration pathway (Lorente-Cánovas et al., 2012; Moreno-Bravo et al., 2014). This migratory stream is exclusively present in r1 mantle (absent at isthmus proper and rest of rhombomeres) and was characterized as expressing Pax7, a transcription factor whose signal in the whole hindbrain is otherwise restricted to the alar ventricular zone (Ju et al., 2004). Thus, the observations in amphibians suggest that at least in the case of the interpeduncular nucleus, both the Pax7 expression and the migratory routes seem conserved in tetrapods.

During mouse development, Pax6 is expressed in the rhombic lip that gives rise to cerebellar granule cells and the precerebellar nuclei (Engelkamp et al., 1999; Fink et al., 2006). Pax6 is also expressed in cerebellar granule cell precursors in chicken (Gilthorpe et al., 2002), *Pleurodeles* (Joven et al., 2013a,b), *Xenopus* (Bandín et al., 2013, 2014), zebrafish (Wullimann and Rink, 2001), and the shark *S. canicula* (Rodríguez-Moldes et al., 2008). The absence of rhombic lip-derived cerebellar and precerebellar systems in lampreys has been related to the lack of Pax6 expression in the rhombic lip (Murakami et al., 2001). Throughout embryonic development, in gnathostomes Pax6 cells form a continuous column along the basal rhombencephalon and spinal cord ventricle (Stoykova and Gruss, 1994; Murakami et al., 2001; Rodríguez-Moldes et al., 2011; Duan et al., 2012). In contrast, agnathans lack Pax6 expression in r4 during the embryonic development (Murakami et al., 2001; Derobert et al., 2002).

Finally, Pax6 cells are also present in the rhombencephalic alar plate in mice, including regions equivalent to the cochlear/vestibular nuclei, in the nucleus of the solitary tract and in the dorsal column nucleus (Stoykova and Gruss, 1994; Duan et al., 2012), and similar observations have been made in the rhombencephalon of amphibians (Joven et al., 2013a,b). Interestingly, it has been suggested that Pax6 is involved in the specification of subtypes of hindbrain neurons (Osumi et al., 1997) and is currently determined as crucial factor in the segmental organization of the early hindbrain (Kayam et al., 2013).

## CONCLUSION: EVOLUTIONARY CONSERVATIVE Pax6 AND Pax7 GENOARCHITECTURE

**FIGURE 9:**
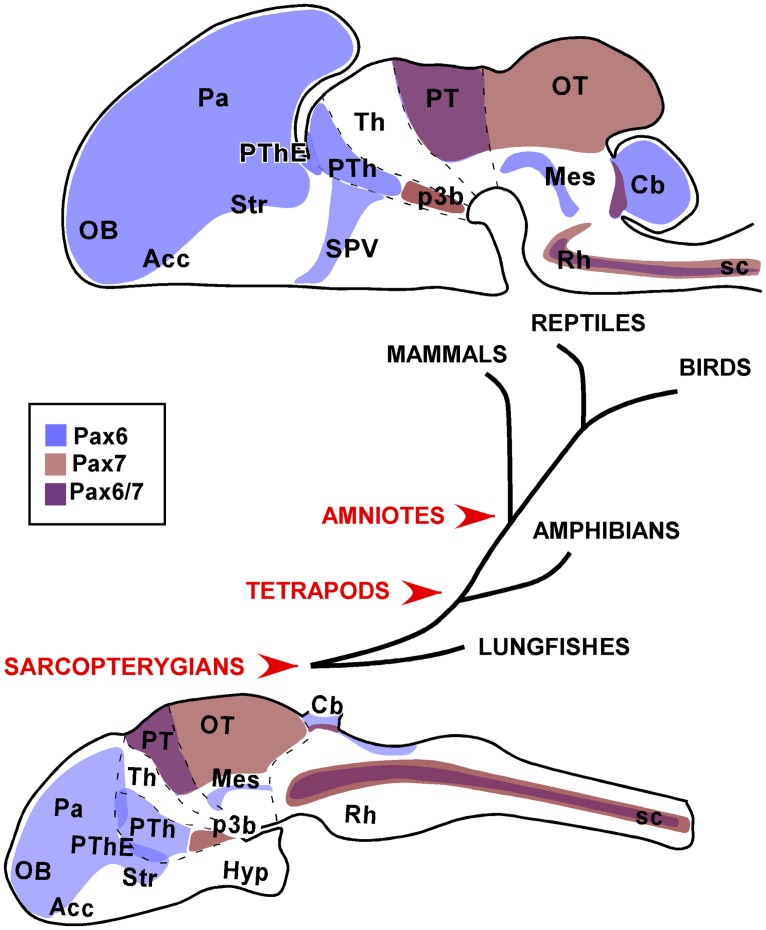
**Schematic representations of the distinct neuroanatomical regions that were consistently observed with Pax6 and Pax7 expression in the brain of the species studied.** The drawings correspond to sagittal views of the brains of a postnatal amniote (upper drawing) versus a juvenile anamaniote (lower drawing).

Comparisons between the patterns of Pax6/7 expression in the developing and adult CNS of vertebrates have shown that they are overall evolutionary conserved (**Figure 9**). The high-resolution immunolocalization of these transcription factors has provided crucial guides for the identification of distinct brain structures and anatomical boundaries across species and, in particular in the developing brain, which could be better understood using the neuromeric model of the brain for the interpretation of topological homology. Thus, Pax expression patterns can be used to support homologous brain regions through vertebrates.

Especially, the present comparative analysis has shown that for the striatal derivatives in the telencephalon of all vertebrates, Pax6 expression results very useful in the identification of the nucleus accumbens and central amygdala, showing in both cases a very conserved expression pattern. Also in the telencephalon, the Pax6 expression in the psb and dorsal striatum is outstandingly conserved and suggests that the migration of Pax6 cells from these regions to adjacent amygdaloid territories might also be a conserved feature. Thus the differences and similarities in the arrangement of these regions could reflect differences in the evolution of the amygdaloid complex or related territories. Moreover, differences in the Pax6 expression are evident in the telencephalon, such as those observed in bed nucleus of the stria terminalis, which only in amniotes possesses Pax6 striatal expressing cells. In addition, a noticeable difference between anamniotes and amniotes is found in the Pax6 expression in the telencephalic–hypothalamic boundary, only detected in amniotes.

In the diencephalic prethalamic eminence Pax6 is detected in the ventricular region in all species studied and additional studies will clarify whether the contribution of Pax6 cells from this region to the amygdaloid complex is a shared feature of vertebrates. In any case, the highly conserved Pax6 expression in the prethalamic eminence suggests that it is likely to be involved in crucial events during the prosencephalic organization.

The conserved Pax7 expression from very early stages of development in the basal plate of p3 is characterized by the ventricular expression of Pax7 and only subsequently is detected in the mammillary area, including the subthalamic nucleus, but not in the ventricular zone (present results). This would maintain the controversial issue of the origin of the subthalamic nucleus from the basal part of p3 (García-López et al., 2009; present results) or from an actual hypothalamic region in the caudal basal region (Jiao et al., 2000; Puelles and Rubenstein, 2003; Martin et al., 2004; Skidmore et al., 2008; Puelles et al., 2012b).

The expression patterns of Pax7 and Pax6 during development and postnatally in the optic tectum (superior colliculus) and the mesencephalic tegmentum, respectively, are among the most constant across all vertebrate classes. The spatiotemporal Pax7 expression supports its importance in the tectal maturation and in the maintenance of specific neuronal functions. In turn, the Pax6 expressing tegmental band might be implicated in crucial event during the mesencephalic organization, especially in the alar/basal definition explaining its high degree of conservation.

In the rostral rhombencephalon, it is of particular interest the outstandingly conserved expression pattern of Pax7 observed in r1 and, in particular, in the interpeduncular nucleus. The common observations in amniotes and anamniotes support that in all vertebrates Pax7 cells participate in the formation of this nucleus, and also the migratory routes from the alar ventricular to the nucleus would be largely similar.

In summary, the Pax genes studied are generally expressed in the ventricular zone of restricted regions in the CNS during early stages and, as development proceeds, the expression changes from these mitotic germinal zones to become distributed in cell groups that in some cases, more than other transcription factors, maintain the expression through adulthood. Such changes have led to suggest different roles for these Pax molecules in *regionalization and subdivision* of the nervous system during early stages, and the differentiation of specific cell populations during late stages (Kawakami et al., 1997; Hsieh and Yang, 2009; Sansom et al., 2009). Recently, it has been demonstrated that the deletion of Pax6 in the subependymal zone causes the progeny of adult neural stem cells to convert to the ependymal lineage while migrating neuroblasts convert to different glial lineages, revealing a neurogenic effect at the maturation stage (Ninkovic et al., 2013). The retained expression, in many cases, in the adult brain is also interesting since these Pax members have been commonly related to neurogenesis and regenerative events. Thus, the widespread presence of Pax6 and Pax7 in distinct and discrete territories might provide a scaffold for migrating processes to gain the adult final brain organization. The spatiotemporal sequences of Pax expression provide indirect evidences of putative *migratory routes*, some of which have already been demonstrated, but not in all models. Those include migrations in the olfactory bulbs, across the psb, along a rostral migratory stream in the telencephalon, from the basal diencephalon to the mammillary hypothalamus, from alar to basal territories in the hindbrain, and across rhombomeric boundaries. The study of all of these migratory processes would constitute a very interesting future research.

## AUTHOR CONTRIBUTIONS

All authors had full access to all the data in the study and take responsibility for the integrity of the data and the accuracy of the data analysis. Nerea Moreno and Agustín González devised the study. Nerea Moreno, Alberto Joven, Sandra Bandín, Jesús M. López, and Ruth Morona performed all the experiments in the different vertebrate classes. Nerea Moreno, Jesús M. López, and Agustín González were the primary contributors to the data analysis. Nerea Moreno, Alberto Joven, and Sandra Bandín led the figure preparation and wrote the majority of the article, further completed and edited by Nerea Moreno and Agustín González.

## Conflict of Interest Statement

The authors declare that the research was conducted in the absence of any commercial or financial relationships that could be construed as a potential conflict of interest.

## ABBREVIATIONS

Acc: nucleus accumbens
ap: alar plate
Cb: cerebellum
CeA: central amygdala
DP: dorsal pallium
EA: extended amygdala
Gc: central rhombencephalic gray
*G.g.*: *Gallus gallus*
hyp: hypophysis
Hypo: hypothalamus
Ip: interpeduncular nucleus
lfb: lateral forebrain bundle
LP: lateral pallium
Ma: mammillary region of hypothalamus
MeA: medial amygdala
Mes: mesencephalon
*M.m.*: *Mus musculus*
MP: medial pallium
*N.f.*: *Neoceratodus forsteri*
OB: olfactory bulb
oc: optic chiasm
OT: optic tectum
Pa: pallium
Pn: pontine nucleus
*P.s.*: *Pseudemys scripta*
psb: pallio-subpallial boundary
PT: pretectum
PTh: prethalamus
PThE: prethalamic eminence
*P.w.*: *Pleurodeles waltl*
p3b: basal part of prosomere 3
Rh: rhombencephalon
Rm: middle reticular nucleus
Rs: superior reticular nucleus
Rt: reticular formation
SC: superior colliculus
sco: subcommissural organ
Se: septum
SPV: supraoptoparaventricular hypothalamic region
Str: striatum
tg: mesencephalic tegmentum
tgd: dorsal mesencephalic tegmentum
Th: thalamus
Ts: torus semicircularis
VP: ventral pallium
*X.l.*: *Xenopus laevis.*
